# Paratuberculosis control: a review with a focus on vaccination

**DOI:** 10.1186/1476-8518-9-8

**Published:** 2011-10-31

**Authors:** Felix Bastida, Ramon A Juste

**Affiliations:** 1 Vacunek, Bizkaiko Teknologia Parkea, Ibaizabal Bidea 800, Derio 48160, Bizkaia, Spain; 2 NEIKER-Tecnalia, Department of Animal Health, Berreaga 1, 48160 Derio, Bizkaia, Spain

**Keywords:** Mycobacteria, paratuberculosis, cattle, sheep, goats, vaccine, protection, production effects, epidemiological effects, pathogenetic effects

## Abstract

*Mycobacterium avium *subsp. *paratuberculosis *(MAP) infection causes in ruminants a regional chronic enteritis that is increasingly being recognized as a significant problem affecting animal health, farming and the food industry due to the high prevalence of the disease and to recent research data strengthening the link between the pathogen and human inflammatory bowel disease (IBD). Control of the infection through hygiene-management measures and test and culling of positive animals has to date not produced the expected results and thus a new focus on vaccination against this pathogen is necessary. This review summarizes all vaccination studies of cattle, sheep or goats reporting production, epidemiological or pathogenetic effects of vaccination published before January 2010 and that provide data amenable to statistical analyses. The meta analysis run on the selected data, allowed us to conclude that most studies included in this review reported that vaccination against MAP is a valuable tool in reducing microbial contamination risks of this pathogen and reducing or delaying production losses and pathogenetic effects but also that it did not fully prevent infection. However, the majority of MAP vaccines were very similar and rudimentary and thus there is room for improvement in vaccine types and formulations.

## Introduction

Paratuberculosis poses a big challenge to Veterinary Medicine and in particular to ruminant production. Since the first description of the disease in 1895 in a cow from Oldenburg, Friesland, its etiological agent, *Mycobacterium avium *subsp. *paratuberculosis *(MAP), has been shown to cause the disease in the majority of wild and domestic ruminant species [1,2]. This microbe is also present in many other hosts as well as the environment [3,4]. Even though the most important mycobacterial infection in animals, bovine tuberculosis, has been successfully controlled in nearly all developed countries, the other important mycobacterial infection, paratuberculosis, remains an unsolved problem for the veterinary scientific community still incapable of reaching a consensus on the better way to deal with it. This is so despite large control efforts in different countries during the past three decades.

The mounting evidence showing that MAP is a factor in the pathogenesis of human inflammatory bowel disease (IBD) has increased the pressure to overcome this challenge. In spite of this, most of the undertakings are nevertheless based on the old principle that the only way to control an infectious disease is to eradicate its agent. This principle has worked well for some acute infections in times of survival struggle and profligate use of means but is increasingly difficult to apply because of demonstrated lack of efficacy and sustainability philosophy [5,6]. We are no longer faced with a live or death dilemma due to infectious diseases, but we have to deal with a need to increase productivity for the sake of improved and prolonged use of scarce resources. From this perspective, it is necessary to simultaneously exploit the three classical main approaches to eradicate or reduce the impact of paratuberculosis in herds or flocks. These are: 1) to introduce management changes to decrease the transmission of MAP, 2) to apply test and cull practices to eliminate the sources of infection, 3) to vaccinate replacers in order to increase their resistance to infection. The advantages and drawbacks of these strategies will be briefly examined.

### Management measures to decrease transmission of MAP

Management changes to reduce the transmission rate are widely accepted strategies that are compatible with all other approaches [7]. Furthermore, these changes have other positive side effects on farm productivity. Management measures focus mainly on avoiding contact between infected and susceptible young animals [8]. These measures include separating offspring from dams immediately after birth, feeding calves paratuberculosis-free colostrum supplement and milk replacement, raising replacement heifers in separate locations, avoiding manure fertilization of fields where replacement heifers grace, improving general farm hygiene, and eliminating practices that can bring infected foods or materials in contact with susceptible animals. In practice, it implies duplication of facilities and equipment, and meticulous working procedures. Also, another very important factor in the spread of paratuberculosis, which complicates the control of this disease through management measures, is the ability of MAP to survive in the environment for around one year [9,10]. Given the different settings and economic constraints of each individual farm, control measures may greatly vary form farm to farm. In addition, control measures should not be neglected when new animals are brought into a herd. Microbiological and serological results of all new animals, as well as, the paratuberculosis status and history of the herd of origin should always be taken into account before introducing new animals into the farm.

Although these measures might be viable for large dairy farms, the required changes may not be economical for many small dairy farms and are probably impossible to implement in beef cattle and sheep operations due to costs and disrupting effects. Moreover, these measures usually yield no immediate results and are easily abandoned when other productive constraints become more pressing [11]. In summary, this type of strategy has low engaging force and has little chance of being widely and successfully implemented in a whole region.

### Culling strategies to eliminate sources of infection

Three variants of the testing and culling strategy prevail depending on the diagnostic method used to detect infected animals: fecal culture, ELISA or Polymerase Chain Reaction (PCR). The slow turn around rate or the low sensitivity of some of these test are the major problems in the efforts to control the disease [12].

### Fecal culture and culling

It is generally accepted that this method detects infected animals first and is the most sensitive method [13,14]. Since it is based on identifying the agent when it is shed into the environment, culling these animals has a direct effect in preventing new infections. Fecal positive animals will also become clinical cases, and, therefore, the most visible effect of culling them is that clinical cases quickly disappear. The main problem with this approach is that the laboratory test is expensive, requires specialization, and its results are not available for several weeks or even months. As a result, progress in control of the disease is slow and often rather disappointing since positive animals keep on appearing over the years even after periods of negative results and absence of clinical cases. Its use for sheep and goats is prohibitively expensive unless it is carried out in pools. Another problem with this approach in farms heavily contaminated with MAP or in farms with super-shedders (animals that excrete 10,000 to 10 million MAP bacteria per gram of manure)[15] is the elimination of uninfected animals that give positive MAP results just because they are passing MAP bacteria through their gastrointestinal tract. This problem also affects PCR and culling strategy.

### ELISA and culling

The ELISA test for paratuberculosis is generally considered to be highly specific, but of low sensitivity [14]. ELISA's simplicity, speed, low cost, and potential for automation makes it an ideal tool for laboratory diagnostic work [16]. The problems with ELISA test are that it has not yet been well studied how it will perform to control the disease and that the minimal sensitivity to reach eradication in a reasonable period of time is not guaranteed. In the best case scenario, inferring from the experience with fecal culture it can be assumed that ELISA testing and culling, if done often enough, will prevent the appearance of clinical cases, and slightly decrease the transmission risk. Additional problems with paratuberculosis ELISA are that sample handling appears to affect substantially the results of the test [17] and that the different commercially available diagnostic kits have very different efficacies [18,19], which therefore, can severely affect control programs. Given its costs are low and the results are obtained in less than a week, it is more easily accepted when positive results keep trailing along time since it is always possible to intensify control by testing more frequently. The regional ELISA specific strategies implemented up to now are rather complex and still not proven successful.

### PCR and culling

The new type of strategy, albeit sparsely implemented, is the combination of PCR analysis of feces and culling of positive animals. In theory, this strategy should detect animals early in the infection process before antibodies are developed, and thus can quickly reduce the overall bacterial burden in the farm. However, the costs and the requirement of specialized personnel are major drawbacks of this technique. Until recently costs of PCR were extremely high for its use in animal health diagnostics. Dramatic reductions in reagent prices accompanied by improvements in technique sensitivity and especially in efficient high-throughput processing of samples and extraction of nucleic acids have made this approach a valuable strategy due to its high specificity, good sensitivity, and fast turnaround time [20,21]. The majority of paratuberculosis PCR detection tests are based on the detection of IS900 sequence, which has the benefit of multiple copies of target DNA per bacteria (higher sensitivity) but the disadvantage of a lower specificity since a few environmental mycobacteria also contain this insertion sequence. Other tests use MAP specific single copy genes (i.e. F57, 251) with theoretically lower sensitivity but higher specificity [22,23]. Multiplex PCRs, using combinations of target genes, have also been reported [24]. PCR has the additional benefit over the ELISA technique that, like fecal culture, it can provide quantitative bacterial content results, and thus high shedders and medium shedders can readily be identified and eliminated. Recently in the Netherlands, fecal culture has been replaced by a PCR based test in the Dutch paratuberculosis control program. As with the ELISA and culling strategy, PCR and culling is not yet proven in the field, however, a new study by Lu et al has shown that the use of faster detection tests such as PCR might be important in farms with poor management [25].

### Vaccination

Vaccination, as a control measure for paratuberculosis, is probably the less accepted strategy although it is or has been used in all countries with substantial problems with this disease [26,27]. It is a highly cost-efficient strategy, which clearly prevents the appearance of clinical cases if done properly [27]. Vaccination strategies have been widely implemented for sheep in different countries with great success [27]. The main drawback to vaccination is that, since vaccines used in the field are not DIVA (differentiating infected from vaccinated), it can interfere with serological diagnosis of paratuberculosis and tuberculosis infections. Thus MAP vaccination might not allow eradication of the disease and it can interfere with national tuberculosis eradication programs. The latter is in fact the major hurdle affecting MAP vaccine approval for cattle by medical and agricultural authorities all over the world and the major deterrent for pharmaceutical companies to design new MAP vaccines for cattle. The most widely used tuberculosis diagnostic test in cattle is the single intradermal tuberculin test, and some cattle vaccinated with the currently available ovine or experimental MAP vaccines will become positive to this test. According to legislation in many countries, these animals are banned from international trade and should be slaughtered unless it can be proved that they are not infected with tuberculosis. New tuberculosis immunological diagnostic test, such as the gamma interferon release assay or the Enferplex™ TB assay, could help in the differentiation between MAP vaccinated and tuberculosis infected animals, but, improvements of these test might be required, since interference with tuberculosis diagnosis can still occasionally occur in MAP infected animals [28]. However, a modification of the single intradermal tuberculin test, the comparative intradermal tuberculin test, could solve the interference problem in the vast majority of cases. This test, which has been available for many years and is actually an official tuberculin test according to the OIE and EU legislation, consists of the simultaneous intradermal injection in two different sites of tuberculins from *Mycobacterium bovis *(PPDbov) and *Mycobacterium avium *subsp. *avium *(PPDav). Higher reactivity to the avian tuberculin indicates infection or vaccination with avian type mycobacteria and allows to rule out mammal tuberculosis infection according to standardized criteria.

An additional drawback to MAP vaccination, which at least in sheep appears not to be of economical relevance [29], is the granulomatous lesion at the injection site produced by most oil-based bacterin vaccines.

In summary, there are several strategies for paratuberculosis control, but there is no generalized consensus on which one or which combination of strategies should be the standard approach. In our opinion, this is in part due to the fact that paratuberculosis control programs emphasize too heavily MAP eradication.

### Pathogenic background

#### MAP distribution

If we take a general view of our knowledge on paratuberculosis, we should point out that MAP is not a classical infectious agent fully complying with Koch's postulates. Indeed, we know that many experimental infections fail to establish the infectious agent in the intestinal tissue and to cause the disease [30-33]. We also know that frequently the initial focal lesions do not progress to clinical stages. More recent evidence has revealed that it is not rare for herds with no clinical history of paratuberculosis and even with a history of negative fecal culture to occasionally show positive fecal culture results [34]. In addition, recent studies on paratuberculosis prevalence have revealed that as many as 60% of some national herds are actually infected [35]. Finally, Pickup and collaborators have shown that MAP is present in the environment at a previously unsuspected high frequency [4]. All this evidence indicates that MAP might be a necessary, but not a sufficient cause of paratuberculosis. Under these conditions, we should therefore ask ourselves: Is paratuberculosis eradication a realistic goal? Is it necessary? Is it profitable for the society in general? Answers to these questions are not readily available because we lack accurate information on the actual distribution of MAP and its potential impact on human health. Reviewing aspects of the pathogenesis and epidemiology may lay the grounds on which control alternative(s) to choose.

#### Forms of infection

**Figure 1:**
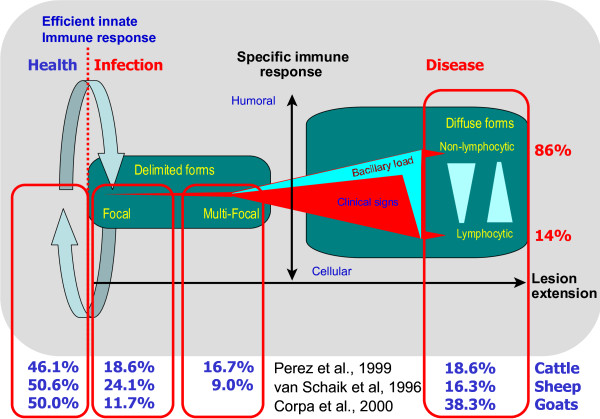
**Immunopathological model of paratuberculosis**. Continuous exposure of animals to MAP results in a dynamic balance where infection never gets established or is controlled by an efficient innate immune response in about half of the farm population, while in the other half it progresses to subclinical delimited focal or multifocal forms and, in a smaller fraction, to diffuse lymphocytic (cellular or Th1 type) or non-lymphocytic (humoral or Th2 type) forms that will result in open clinical disease.

Multiple forms of infection can be observed in MAP infected animals. The form present in an animal will not only depend on the progression of the infection or stage of the disease, but also on many other factors including an individual's genetic resistance or susceptibility to the pathogen, age at the time of infection, and previous exposure to other environmental mycobacteria. On Figure 1, we illustrate the balance between the infection and the animal's immune system and their corresponding forms of infection. According to different studies, about 46% of cattle, 51% of sheep, and 50% of goats in a MAP-contaminated environment do not show any signs of infection [36-38]. Since these animals live in a heavily contaminated environment, they must continuously be exposed to MAP, and, therefore, they either prevent the infection or very quickly clear up the establishment of local infection foci. Because it is not rare for such animals to carry MAP and plenty of experimental evidence has shown that administration of large amounts of MAP not always results in the development of a full blown infection, quite the opposite frequently produces very regressive lesions, the more likely explanation is that there is a balance between MAP and the host that in about half of the exposed individuals results in containing the infection (Figure 1). Beyond this balance point there are also different stages of infection. About 19% of cattle, 24% of sheep and 12% of goats carry an infection which is very focal and delimited. Around 17% and 9% of cattle and sheep, respectively, have multifocal forms. Of the animals presenting diffuse forms, approximately 19% of cattle, 16% of sheep and 38% of goats develop into diffuse forms which lead to animals showing clinical signs and to their death.

#### Vaccine types

Both live (non-attenuated and attenuated) and killed whole cell vaccines have been used against paratuberculosis. In a few cases, subunit vaccines consisting of sonicated bacteria, bacterial cell fractions or recombinant MAP antigens have been used but they have shown a much lower degree of protection [39,40]. More recently, DNA vaccines, consisting of the inoculation of mammalian expression vectors containing MAP genes have also been used in mice, humans and sheep but not in cattle [41-47]. Most MAP vaccine formulations have been based on mycobacteria and a water-in-oil emulsion (olive, mineral, liquid, paraffin, etc). Some have also an irritant like pumice powder in order to increase and stimulate the local inflammatory response, and therefore enhance the immunogenicity of the vaccine. The goal of these vaccines is to establish a focus of inflammation where the antigens can permanently stimulate the host immune system. Under this principle, it would not be necessary to revaccinate animals because the slow liberation of antigens from the vaccination site keeps on stimulating the immune system, at least during the period before the age of initial clinical disease presentation.

#### Vaccination age

Paratuberculosis vaccines are recommended for exclusive use in very young animals on the grounds that this is necessary to prevent infection and to decrease interfering responses with the diagnosis of tuberculosis. Actually, the experience on animals older than 1 month is rather scarce, however recent studies on the pathogenesis as well as some field data suggest that vaccination of adult or subadult animals might have some management (no need for separate handling, vaccination of only replacers) and therapeutic (stronger humoral and cellular responses) advantages that need to be taken into account [48,49]. More recent evidence form Australian sheep vaccination trials indicate that there might be an age threshold for vaccine efficacy that can be drawn at around 8 months of age [50].

### Reassessment of vaccination results

#### Literature on vaccination

There is an increasing number of vaccination studies in ruminant species focused on different aspects of the use of MAP vaccines including two recent reviews on the topic [51,52]. The most recent review by Rosseels et al. focused mainly on the immunological aspects of MAP vaccination [52]. For the purpose of the present review we have used only vaccinations studies of cattle, sheep or goats reporting production, epidemiological or pathogenetic effects and data that could be used to estimate the reduction rates of damage or contamination. Production effects relate to the losses measured as the frequency of clinical cases or mortality rates. We considered epidemiological effects as the microbiological contamination risks measured by the frequency or amount of MAP isolations in fecal or tissue cultures. And finally, pathogenetic effects pertain to the modification in the course of the disease as measured by the frequency of specific histopathological lesions. Searches of published material before January 2010 were run using three strategies: First, specific searches of combinations of the words vaccination, vaccine and paratuberculosis were run on Current Contents or Pubmed and the hits were screened for articles meeting the conditions stated above. Second, the same combinations of words were used in Google (http://www.google.com) to obtain studies from doctoral dissertations and other sources. Third, literature data on vaccination trials collected over a period of 25 years at NEIKER was also examined systematically. More than half the published studies included in this meta-analysis describe field reports, which actually might give a better view of the whole problem of vaccination, since highly controlled experimental trials might be misleading because of the lack of interferences from field conditions.

The very first report on paratuberculosis vaccination of cattle is that by Vallée and Rinjard in 1926 [53]. It is not until 1960 that a similar vaccine was reported to have been used in sheep [54]. As for goats, although it is known that vaccines have been used in Spain in the 70's, the first written report on its efficacy dates back to 1985 in Norway [26].

#### Paratuberculosis vaccination meta-analysis

Taking worldwide published reports on paratuberculosis vaccination available to us but not restricted to peer-reviewed papers, we have classified the studies according to species (cattle, sheep or goats), and type of evaluation of vaccine efficacy (production, epidemiological or pathogenetic effects). We have kept only those studies were the authors reported either vaccinated versus control group or pre-vaccination versus post-vaccination cohorts in numerical terms. In all, except in one study where a scoring system was used for MAP isolation, results were presented as the frequency of positive/affected individuals over total animals in the study. We have not been overly critical on the criteria applied by authors, but instead we have assumed that they knew well the disease and that their study design was sound.

All data have been transformed into a reduction percent calculated as the frequencies difference divided by the frequency in the control group. For each category of species and type of evaluation, we have calculated a size-weighted reduction average for the whole set of studies in that category. The same size-weighting method has been previously used to calculate a standard deviation in order to define the 95% confidence limits of the estimate [55].

## Results

A total of 118 experiments from 63 reports and 14 countries have been used for the meta-analysis in this review (Tables 1 and 2). The USA was the country with the highest number of studies included (26.3%), followed by New Zealand (14.4%) and then closely by Spain (13.6%). Some countries, such as the USA, have studies throughout the years, however, interest in MAP vaccination studies change among countries. For example, early large studies in the UK and France, gave way to studies in The Netherlands, New Zealand, Australia and Spain. This pattern might reflect MAP prevalence levels and research funding priorities in the different countries, but most likely it is also biased by administrative regulations limiting the availability of a successful commercial vaccine for sheep and goats (Gudair™), which is being widely used in countries with large sheep populations. 45 experiments were conducted in cattle, 49 in sheep, and 24 in goats (Table 2). Apart from the studies where small ruminants were used either because they were the target species of the commercial vaccine or because they are an easier to handle and a less costly animal model, there is a relation between the type of animal used in the study and the main livestock in the country.

**Table 1:** Countries where the vaccination experiments* used in the meta-analysis were carried out.

Country	Number of Experiments	Percent
Australia	12	10.2
Denmark	1	0.8
France	5	4.2
Germany	1	0.8
Greece	6	5.1
Hungary	1	0.8
Iceland	2	1.7
India	4	3.4
Netherlands	12	10.2
New Zealand	17	14.4
Norway	1	0.8
Spain	16	13.6
United Kingdom	9	7.6
United States	31	26.3
Total	118	

* An experiment is defined as vaccine trial whose results are measured according to one of the three outcome variables: clinical signs, MAP isolation, gross or microscopic lesions.

**Table 2:** Experiments and reports used for the meta-analysis.

Species	Experiments	Reports*
Cattle	45	33
Sheep	49	21
Goats	24	9

* A report is a publication or communication that might contain results of one or more experiments.

Half of the studies are field trials where animals were naturally exposed to MAP. In these studies, results were assessed either by comparison between initial prevalence before vaccination, and final prevalence some time post-vaccination, or by following up a matched group within the same herd or flock. The later type of studies, when the control group is housed with vaccinated animals, frequently underestimates the positive effects of vaccination, because as herd immunity increases, bacterial shedding into the environment is reduced and thus the probability of a natural infection in the control group is also reduced. In three experiments the assessment was done using control unvaccinated herds, and one consisted of a questionnaire on clinical incidence in farms before and after using vaccination.

Tables 3, 4 and 5 summarize the results of all vaccination experiments used for the meta-analysis. Less than a third of them are not standard peer review journal publications (Doctoral Dissertations, non-peer review magazines, conference proceedings, bulletin reports, memoranda, or other types of documents). Some appear to be advances of results that have been published later. Since the information is different, we have treated them as individual experiments, although we were aware that they might introduce a bias to underestimate vaccination positive effects, particularly regarding culture results because of their lack of time span for the vaccine to make its mid- to long-term effects.

**Table 3:** Production effects (Paratuberculosis clinical cases or mortality rates).

Vaccine				Country and reference	Year	Number of animals	Age at vaccination	Reduction (%)	Type of trial
						
Name/Laboratory	Type	Strain/Antigen	Adjuvant						
				**Cattle**					

NCV	Live	6 strains	Oil	U.S.A. [65]	1935	20	1 m	100,00	E, MC, CC
Weybridge	Live	316F	P/O/P	U.K. [66]	1959	63401	1 m	93.45	F, IF, CC
Weybridge	Live	316F	P/O/P	U.K. [67]	1964	2440	1 m	98.36	F, IF, CC
Weybridge	Live	316F	P/O/P	U.K. [68]	1965	84	1 w	46.67	E, MC, CC
Weybridge	Live	316F	P/O/P	U.K. [69]	1982	150000	1 m	99.06	F, IF, CC
Fromm	Killed	*M.a.a *strain 18	Oil	U.S.A. [70]	1983	48	1 m	35.29	F, MC, CC
-	Live	316F	P/O/P	France [61]	1988	902	1 m	87.34	F, IF, CC
-	Live	316F	P/O/P	France [61]	1988	1037	1 m	97.22	F, IF, CC
Lelystad	Killed	-	Oil	Netherlands [59]	1988	851	1-24 m	87.05	F, IF, CC
Lelystad	Killed	-	Oil	Netherlands [71]	1992	61050	1 m	91.82	F, MC, CC
NCV	Killed	-	Oil	Netherlands [72]	1994	337	1 m	79.01	F, IF, CC
NCV	Killed	-	Oil	Netherlands [37]	1996	573	1 m	68.14	F, CC

**Average**								**96.02 ± 0.01**	

**Sheep**									

NCV	Live	316F	Oil Paraffin	Greece [73]	1988	1448	1 m	76.14	F, MC, TM
NCV	Live	316F	Oil Paraffin	Greece [73]	1988	5526	Adults	28.74	F, MC, TM
Lio-Johne	Live	316F	Oil	Spain [74]	1993	1201	Adults	78.29	F, MC, CC
Lio-Johne	Live	316F	Oil	Spain [75]	1995	570	1 m	52.55	F, MC, TM
Weybridge	Live	316F	P/O/P	U.K. [76]	1993	830	Adults	89.86	F, IF, CC
Neoparasec & NCV	Live & Killed	316F -	Oil Oil	Spain [77]	1995	857	Adults	54.55	F, IF, CC
Neoparasec	Live	316F	Oil	New Zealand [78]	2000	28	1-1.5 m	71.43	E, MC, CC
Gudair	Killed	316F	Oil	Australia [79]	2003	8000	3, 8 m, 2 y	87.50	F, IF, mort rate
Gudair	Killed	316F	Oil	Australia [80]	2004	1200	1-4 m	90.00	F,MC, mort reduction
Gudair	Killed	316F	Oil	Australia [34]	2006	400	1-3 m	91.25	F, MC, TM
Gudair	Killed	316F	Oil	New Zealand [81]	2009	65	4 m	78.57	E, MC, CA
NCV	Killed	316F	Lipid-K formulation	New Zealand [81]	2009	65	4 m	57.14	E, MC, CA
NCV	Live	316F	Lipid-K formulation	New Zealand [81]	2009	65	4 m	14.29	E, MC, CA
NCV	Live	316F	Lipid-K formulation	New Zealand [81]	2009	65	4 m	35.71	E, MC, CA

**Average**								**67.57% ± 0.35**	

**Goats**									

NCV	Live	316F	Oil Paraffin	Greece [73]	1988	2178	1 m	82.78	F, MC, TM
NCV	Live	316F	Oil Paraffin	Greece [73]	1988	7773	Adults	34.52	F, MC, TM

**Average**								45.08 ± 0.39	

NCV: non-commercial vaccine; Weybridge: Central Veterinary Laboratory, Weybridge, UK; Fromm: Fromm Laboratories, Grafton, Wisconsin USA; Lelystad: Central Veterinary Institute, Lelystad, The Netherlands; Lio-Johne, Ovejero, Spain; Neoparasec: Neoparasec^®^, Merial; Gudair: Gudair^®^, CZ Veterinaria/Pfizer; P/O/P Paraffin, Olive Oil, Pumice Stone Powder; y: year(s); m: month(s); w: week(s); d: day(s); F: Field trial; E: Experimental infection; MC: Comparison to matched controls; IF: Comparison of initial versus final prevalence; TM: Total mortality; CC: clinical cases; NVH: Comparison to non-vaccinating herds.

**Table 4:** Epidemiological effects (*Mycobacterium avium *subsp. *paratuberculosis *isolation from faeces or tissues).

Vaccine				Country and reference	Year	Number of animals	Age at vaccination	Reduction (%)	Type of trial
						
Name/Laboratory	Type	Strain/Antigen	Adjuvant						
**Cattle**									

NCV	Live	6 strains	Oil	U.S.A. [65]	1935	20	1 m	-14.29	E,TC
Weybridge	Live	316F	P/O/P	U.K. [68]	1965	84	1 w	11.54	E, MC, TC
Weybridge	Live	316F	P/O/P	Australia [82]	1971	82	1 m	24.18	F, IF,MC, TC
NCV	Live	avirulent	P/O/P	U.S.A. [83]	1974	16	16 d	81.47	E, MC, FC
NCV	Live	avirulent	P/O/P	U.S.A. [83]	1974	16	16 d	0.00	E, MC, TC
Fromm	Killed	*M.a.a *strain 18	Oil	U.S.A.[70]	1983	158	1 m	79.28	F, MC, FC
Fromm	Killed	*M.a.a *strain 18	Oil	U.S.A. [70]	1983	3060	1 m	99.11	F, IF, FC
NCV	Live	316F	Oil	Denmark [84]	1983	5446	1 m	92.90	F, MC, FC
Lelystad	Killed	-	Oil	Netherlands [71]	1992	2065	1 m	-21.25	F, IF, FC
NCV	Live	316F	P/O/P	France [85]	1992	22988	1 m	81.68	F, IF/MC, FC
Phylaxia	Killed	5889 Bergey	Oil	Hungary [86]	1994	2738	1 m	94.70	F, IF, FC
NCV	Killed	-	Oil	Netherlands [72]	1994	499	1 m	-36.72	F, IF, TC
NCV	Killed	-	Oil	Netherlands [37]	1996	573	1 m	13.34	F, IF, TC
Mycopar	Killed	*M.a.a *strain 18	Oil	U.S.A.[87]	2000	372	< 35 d	71.43	F, MC, FC
NCV	Killed	-	Oil	Netherlands [88]	2001	4452	1 m	33.83	F, NVH, FC
Neoparasec	Live	316F	Oil	Germany [89]	2002	521	1 m	86.87	F, MC, FC
Mycopar	Killed	*M.a.a *strain 18	Oil	U.S.A. [58]	2003	10	7 d	-28.00	E, MC, FC, TC
Mycopar IL-12	Killed	*M.a.a *strain 18	Oil	U.S.A. [58]	2003	10	7 d	32.00	E, MC, FC, TC
Mycopar	Killed	*M.a.a *strain 18	Oil	U.S.A. [58]	2003	14	8 d	40.00	E, MC, FC, TC
Mycopar IL-12	Killed	*M.a.a *strain 18	Oil	U.S.A. [58]	2003	14	8 d	23.60	E, MC, FC, TC
Silirum	Killed	316F	Oil	Spain [90]	2005	14	2 m	62.50	E, MC, TC
NCV	Rec	Hsp70	DDA	Netherlands [39]	2006	20	1 m boost 11 m	37.50	E, MC, FC
Mycopar	Killed	*M.a.a *strain 18	Oil	U.S.A. [91]	2006	213	< 35 d	77.12	F, MC, FC
NCV	Rec	MAP (85A, 85B, 85C, SOD)	MPLA +/- IL12 RIBI	U.S.A. [92]	2008	24	5-10 d	41.67	E, MC, FC, TC
Silirum	Killed	316F	Oil	U.S.A. [93]	2009	12	14 d	84.61	E,MC,TC
Silirum	Killed	316F	Oil	Spain [49]	2009	371	all ages	68.20	F, IF, FC, FP

**Average**								**72.55 ± 0.29**	

**Sheep**									

NCV	Killed	101 sheep & VB/4 cattle	Oil	U.K. [94]	1961	44	1 m	52.63	E, MC, TC
NCV	Killed	-	Oil	U.K. [95]	1962	126	1 m	29.05	E, MC, TC
Lio-Johne	Live	316F	Oil	Spain [74]	1993	1201	Adults	80.01	F, MC, TC
Neoparasec	Live	316F	Oil	Spain [96]	1994	13	2 m	38.89	E, MC, TC
Neoparasec & NCV	Live & Killed	316F -	Oil	Spain [77]	1995	97	Adults	-10.95	F, IF, TC
NCV	Killed	-	Oil Paraffin	Greece [97]	1997	226	1 m	93.27	F, MC, FC
Neoparasec	Live	316F	Oil	New Zealand [78]	2000	28	1-1.5 m	66.67	E, MC, TP
Gudair	Killed	316F	Oil	Australia [80]	2004	1200	1-4 m	90.00	F, MC, FC
Gudair	Killed	316F	Oil	Australia [98]	2005	-	16 w	52.21	F, IF, FC
Gudair	Killed	316F	Oil	Australia [34]	2006	400	1 m	76.14	F, MC, FC
Gudair	Killed	316F	Oil	Australia [34]	2006	400	1 m	84.15	F, MC, FC
Gudair	Killed	316F	Oil	Australia [99]	2007	998	2-3 m	76.14	F, MC, FC
Gudair	Killed	316F	Oil	New Zealand [81]	2009	62	4 m	25.30	E, MC, FC
NCV	Killed	316F	Lipid-K formulation	New Zealand [81]	2009	63	4 m	36.03	E, MC, FC
NCV	Live	316F	Lipid-K formulation	New Zealand [81]	2009	63	4 m	36.03	E, MC, FC
NCV	Live	316F	Lipid-K formulation	New Zealand [81]	2009	62	4 m	34.09	E, MC, FC

**Average**								**76.42 ± 0.54**	

**Goats**									

Neoparasec	Live	316F	Oil	France [100]	1988	27	1 m	73.08	E, MC, FC
Neoparasec	Live	316F	Oil	France [100]	1988	26	1 m	51.01	E, MC, TC
Fromm	Killed	-	Freund's Complete	U.S.A. [101]	1988	1075	1 m	80.23	F, MC, FC
NCV	Killed	-	Oil Paraffin	Greece [97]	1997	297	1 m	95.57	F, NVH, FC
NCV	Killed	Goat isolate (CWD)	QS21	U.S.A. [102]	2007	20	1-4 w	61.69	E, MC, FC, TC
NCV	Killed	Goat isolate (CWC)	QS21	U.S.A. [102]	2007	20	1-4 w	85.19	E, MC, FC, TC
NCV	Killed	Goat isolate (CWC)	Alum	U.S.A. [102]	2007	20	1-4 w	79.31	E, MC, FC, TC
NCV	Killed	Goat isolate (CWD)	Alum	U.S.A. [102]	2007	20	1-4 w	-57.68	E, MC, FC, TC
NCV	Killed	Virulent Field Strain	Alum	India [48]	2007	55	4-6 m	82.14	E, MC, FC
Gudair	Killed	316F	Oil	India [48]	2007	55	4-6 m	52.38	E, MC, FC
NCV	Rec	MAP (85A, 85B, SOD, 74F)	DDA	U.S.A. [40]	2009	17	5-10 d	87.50	E, MC, TC
NCV	Rec	MAP (85A, 85B, SOD, 74F)	none	U.S.A. [40]	2009	17	5-10 d	37.50	E, MC, TC

**Average**								**79.34 ± 0.89**	

NCV: non-commercial vaccine; Weybridge: Central Veterinary Laboratory, Weybridge, UK; Fromm: Fromm Laboratories, Grafton, Wisconsin USA; Lelystad: Central Veterinary Institute, Lelystad, The Netherlands; Phylaxia: Phylaxia Veterinary Biologicals Company, Budapest; Mycopar^®^: Mycopar Fort Doge/Solvay, USA; Neoparasec: Neoparasec^®^, Merial; Silirum: Silirum^®^, CZ Veterinaria/Pfizer; Lio-Johne, Ovejero, Spain; Gudair: Gudair^®^, CZ Veterinaria/Pfizer; Rec: recombinant; CWD Cell Wall Deficient MAP; CWC Cell Wall Competent MAP; P/O/P Paraffin, Olive Oil, Pumice Stone Powder; y: year(s); m: month(s); w: week(s); d: day(s); F: Field trial; E: Experimental infection; MC: Comparison to matched controls; IF: Comparison of initial versus final prevalence; NVH: Comparison to non-vaccinating herds; TC: Tissue culture; FC: Fecal culture.

**Table 5:** Pathogenetic effects (histopathological lesions).

Vaccine				Country and reference	Year	Number of animals	Age at vaccination	Reduction (%)	Type of trial
						
Name/Laboratory	Type	Strain/Antigen	Adjuvant						
**Cattle**									

NCV	Live	6 strains	Oil	U.S.A. [65]	1935	20	calves	42.86	E, HP
NCV	Live	avirulent	P/O/P	U.S.A. [83]	1974	16	16 d	17.24	E, HP
Lelystad	Killed	-	None	Netherlands [71]	1992	3209	1 m	58.34	F, IF, HP
NCV	Killed	-	Oil	Netherlands [72]	1994	499	1 m	57.23	F, IF, HP
NCV	Killed	-	Oil	Netherlands [37]	1996	573	1 m	58.09	F, IF, HP
Silirum	Killed	316F	Oil	Spain [103]	2005	79	all ages	38.68	F, MC, HP
Silirum	Killed	316F	Oil	Spain [90]	2005	14	2 m	37.50	E, MC, HP

**Average**								**57.54 ± 0.11**	

**Sheep**									

NCV	Killed	-	Oil	Iceland [54]	1960	419	3 m	83.58	F, MC, PM
NCV	Killed	-	Oil	Iceland [54]	1960	24323	3 m	93.55	F, MC, PM
NCV	Killed		Oil	U.K. [95]	1962	126	1 m	52.22	E, MC, HP
Lio-Johne	Live	316F	Oil	Spain [74]	1993	570	1 m	100.00	F, MC, HP
Lio-Johne	Live	316F	Oil	Spain [74]	1993	1201	Adults	53.36	F, MC, HP
Neoparasec	Live	316F	Oil	Spain [96]	1994	13	2 m	64.52	E, MC, HP
Neoparasec	Live	316F	Oil	Australia [104]	1995	475	3 m	82.27	F, MC. HP
Neoparasec & Gudair	Live and Killed	316F	Oil	Spain [77]	1995	135	Adults	-3.03	F, IF, HP,
Neoparasec	Live	316F	Oil	New Zealand [78]	2000	28	1-1.5 m	77.78	E, MC, HP
Gudair	Killed	316F	Oil	Spain [105]	2002	12	1 m	100.00	E, MC, HP
Mycopar	Killed	*M.a.a*. Strain 18	Oil	U.S.A. [106]	2005	178	60-164 d	75.31	F, MC, HP
Neoparasec	Live	316F	Oil	New Zealand [57]	2005	59	2-4 w	68.52	E, MC, HP
AquaVax	Live	316F	saline	New Zealand [57]	2005	58	2-4 w	-2.48	E, MC, HP
Gudair	Killed	316F	Oil	Australia [34]	2006	88	1-3 m	72.70	F, MC, GL, HP
Gudair	Killed	316F	Oil	Australia [34]	2006	307	1-3 m	48.29	F, MC, GL, HP
Gudair	Killed	316F	Oil	New Zealand [81]	2009	62	4 m	75.57	E, MC, HP
NCV	Killed	316F	Lipid-K formulation	New Zealand [81]	2009	63	4 m	37.17	E, MC, HP
NCV	Live	316F	Lipid-K formulation	New Zealand [81]	2009	63	4 m	51.32	E, MC, HP
NCV	Live	316F	Lipid-K formulation	New Zealand [81]	2009	62	4 m	57.56	E, MC, HP

**Average**								**89.70 ± 0.15**	

**Goats**									

NCV	Live	2E/316F	P/O/P	Norway [26]	1985	5535	1 m	97.18	F, IF, PM
Gudair	Killed	316F	Oil	Spain [38]	2000	189	Adults	65.88	F, MC, HP
NCV	Killed	Goat isolate (CWD)	QS21	U.S.A. [102]	2007	20	1 w	34.38	E, MC, HP
NCV	Killed	Goat isolate (CWC)	QS21	U.S.A. [102]	2007	20	1 w	32.03	E, MC, HP
NCV	Killed	Goat isolate (CWC)	Alum	U.S.A. [102]	2007	20	1 w	44.53	E, MC, HP
NCV	Killed	Goat isolate (CWD)	Alum	U.S.A. [102]	2007	20	1 w	-17.19	E, MC, HP
NCV	Killed	Virulent Field Strain	Alum	India [48]	2007	8	4-6 m	75.00	E, MC, HP
Gudair	Killed	316F	Oil	India [48]	2007	8	4-6 m	50.00	E, MC, HP
NCV	Rec	MAP(85A, 85B, SOD, 74F)	DDA	U.S.A. [40]	2009	17	5-10 d	66.67	E, MC, HP
NCV	Rec	MAP(85A, 85B, SOD, 74F)	none	U.S.A. [40]	2009	17	5-10 d	33.33	E, MC, HP

**Average**								**94.79% ± 0.29**	

NCV: non-commercial vaccine; Lelystad: Central Veterinary Institute, Lelystad, The Netherlands; Silirum: Silirum^®^, CZ Veterinaria/Pfizer; Lio-Johne, Ovejero, Spain; Neoparasec: Neoparasec^®^, Merial; Gudair: Gudair^®^, CZ Veterinaria/Pfizer; Mycopar^®^: Mycopar Fort Doge/Solvay, USA; AquaVax; Rec: recombinant; CWD Cell Wall Deficient MAP; CWC Cell Wall Competent MAP; P/O/P Paraffin, Olive Oil, Pumice Stone Powder; y: year(s); m: month(s); w: week(s); d: day(s); F: Field trial; E: Experimental infection; MC: Comparison to matched controls; IF: Comparison of initial versus final prevalence; GL: Gross lesions; HL: Histological lesions.

**Figure 2:**
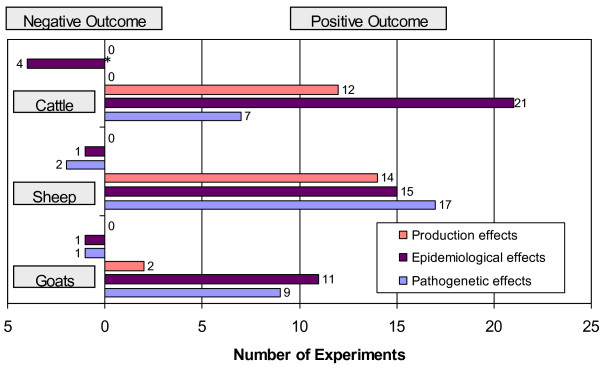
**Types of MAP vaccination experiments used in the meta-analysis**. Graphic representation of MAP vaccination experiments grouped by outcome according to animal species and category (production, epidemiological or pathogenetic effects). Experiments with a negative outcome: bars on the left part of the chart; experiments with a positive outcome: bars on the right hand of the chart. Numbers adjacent to the bars correspond to the number of experiments. *One experiment has a 0% reduction.

The vast majority of studies on all species showed positive reductions in all examined variables (Figure 2), that in cattle resulted in average reductions of 96.0%, 72.6% and 57.5% for production, epidemiological or pathogenetic effects, respectively. In sheep these reductions were of 67.5%, 76.4% and 89.7% and in goats of 45.1%, 79.3% and 94.8%, clearly demonstrating that MAP vaccination works well in all three species. The widest spread in reduction percentages, including several negative reduction rates, was observed with the epidemiological effects variable, which represents culture data. These differences are probably due to inherent aspects of each variable, since frequently the same study that gave negative reduction rates with the epidemiological variable, showed much better reduction results with the other variables, specially for the production effects variable. Most studies reported culture data as positive or negative result and did not include data on quantification of bacterial load in the sample. Thus, vaccinated animals with clinical signs reduction were still infected and excreted bacteria. This would imply that even though the amount of bacterial shedding might have been reduced, the proportion of shedding animals might have not. As a consequence, this would be in agreement with the widely accepted concept that, in general, current MAP vaccines can contain the infection and dramatically decrease clinical signs in a herd, but do not completely clear the infection.

Except for a few cases, vaccination in cattle was applied at early ages, in the first weeks of life, while in sheep more studies included adult sheep. The largest sample size studies, up to 150,000 animals, were done in cattle and preferentially recorded production effects in terms of paratuberculosis culling rates, since measurement of the other variables is much more time-consuming and costly. This is also reflected in the median sample size for the studies that looked at the production variable, 876, 700, and 4975 animals for cattle, sheep and goat studies, respectively, while studies analyzing the epidemiological or pathogenetic variables had median sizes around 100 or less.

The range of study length was between a few months and 16 years covering a period of 74 years. A large increase in sheep studies in the last decade coincided with the availability of the successful small ruminant commercial vaccine Gudair™ and its extended application in Australia and New Zealand. In the majority of the studies (68 experiments) killed vaccines were used. Most experiments used MAP strain 316F from Weybridge, nine used Strain 18 (now known to be *M. avium *subsp. *avium *rather than MAP [56]), and the rest used local isolates or subunit vaccines consisting of recombinant proteins. Not surprisingly, 316F is the most frequently used strain in sheep studies, since the above mentioned commercial vaccine for sheep and goats is based on this strain. Bacterial content varied widely, from 1000 CFU to 3 × 10^9 ^CFU, and from 2.5 mg to 100 mg. The large majority of studies used some type of oily adjuvant (mineral oil, olive oil, liquid paraffin etc.) and in very few cases alum. In one study [57], AquaVax experimental vaccine was used, which contains no adjuvant but saline instead. More recent studies have started using other newer adjuvants such as MPLA, RIBI, cytokines, DDA, QS21, and lipid formulations, some of them with good results.

## Discussion

A wide variation in the efficacy of vaccines was observed, especially in cattle and sheep, where negative reductions are described in some studies. However, the overall results are pretty homogeneous, with very small error ranges due to the large numbers of observations included. In general, vaccine strain or administration route differences do not seem to substantially alter the outcome of vaccination, however, type of antigen formulation or adjuvant appears to have been important in a few experimental studies where different formulations were compared side by side [58].

The goal of this review was to evaluate vaccination as a whole, summarizing the results into a single table for each type of measure used to determine vaccine success. This analysis has revealed that, in average, vaccination has a positive effect. However, since the efficacy figures are rather poor in comparison to vaccines for other microorganisms, it is relevant to at least try to discuss possible reasons for some of the low success rates. In order to simplify, one possible approach is to find an explanation for studies where vaccination performed below the average. Production effect studies where the measurement was total mortality may be considered flawed because in most cases mortality did not differentiate between paratuberculosis and other pathologies, possibly diluting the "vaccine effect". This is evident in the case of sheep and goat trials were adults were considered. Since the more sensitive part of the population might have already died of paratuberculosis before vaccination the remaining animals could be considered more resistant to paratuberculosis, and therefore, less likely to show any effect of vaccine protection. Because young animal studies showed larger effects, this becomes a very likely explanation for low reduction rates. An additional explanation for the poor results could be the fact that, frequently, vaccination programs coincide with the initiation of other control measures making it difficult to assess the real effect of the vaccine on paratuberculosis control.

Under the epidemiological effects analyzed, reduction in the proportion of fecal shedders appears to be one of the measurements showing the widest variability. This happens mostly in small studies or in studies carried out in the Netherlands. Besides the qualitative effects in terms of protection conferred, vaccination should be assessed from another, perhaps even more important standpoint, such as is the reduction of amounts of bacteria shed by vaccinated and non-vaccinated animals.

When considering reduction in pathogenetic effects, it should be pointed out that some of the studies had a very short follow up and that the presence of focal lesions of paratuberculosis are weighted the same as the presence of large areas of affected intestine.

Since the vast majority of the studies show a positive effect, the question as to why vaccination has not been given more opportunities comes out with force. Especially, because for years MAP eradication efforts have only shown very moderate success or straight failure due to their enormous costs and frequent relapses of infected animals. Already in the eighties [59] and nineties [37,60] several studies showed the profitability of vaccination. Over a period of a few years, the economic advantages of vaccination may be up 20 times higher than any testing and culling strategy which, in addition to yielding uncertain results, it results in a much higher economic cost. Other strategies based on certification are compatible with vaccination, and moreover, vaccination might allow a spectrum of other approaches to paratuberculosis control dependant on the financial resources of the farm, region or farmers association, and the actual economic losses sustained by the enterprise. It has been estimated that only a 5% annual clinical incidence of paratuberculosis will justify entering a mixed vaccination and testing and culling strategy [61].

In our opinion, there is a mixture of vested interests on control programs based in testing and culling, simplistic thinking comparing tuberculosis and paratuberculosis, fear of cross-reactions, academic detachment and confusion between ideal objectives and practical needs for the livestock industry. It is clear that the main reason for the opposition to MAP vaccination in cattle has been the problem of expected interference with the diagnosis of tuberculosis and its consequences in trade and national TB programs, however, the availability of an OIE official test -the comparative intradermal tuberculin test- that can very easily solve this problem in the majority of cases, should eliminate this concern on MAP vaccination in cattle. Recent field vaccination trials in cattle with an experimental MAP vaccine (Silirum™, CZV), have shown that less than 0.5% of vaccinated animals will give interference problems when the comparative intradermal tuberculin test is used even if the most restrictive interpretation of results proposed by the OIE is applied (Joseba Garrido, personal communications). The benefits obtained from production increases and reduction in clinical cases of MAP, will largely outweigh the small loss due to culling of these tuberculosis cross-reactive animals. In addition, new plans for the introduction of improved tuberculosis vaccines for cattle [62], will also affect the prospects of MAP vaccination in cattle, since the accompanying DIVA diagnostic test will probably allow for the identification of MAP infected or vaccinated animals.

MAP vaccination concerns in cattle have been further aggravated by the fear of the dairy industry to a crisis of confidence in their products, particularly, if a potential zoonotic link between paratuberculosis and a human disease (IBD/Crohn's disease) is confirmed [63] or if too much discussion and research efforts are focused on this subject. At this moment in which the paratuberculosis scientific community has finally accepted that the key to the paratuberculosis problem might not be eradication, but just control, vaccination offers the solution to this problem, since it not only allows to confine the paratuberculosis problem within the limits of a livestock production issue, while downright calming the worries of farmers, but also provides the perfect cover for doing something against paratuberculosis from a Public Health point of view, without incurring in massive costs. Vaccination might be the beginning of the end of the huge worldwide paratuberculosis problem and might mark the difference between doing nothing and advancing towards global control [64].

## Conclusions

Paratuberculosis control poses a though challenge for farmers and veterinarians. Test and cull strategies can be useful in some settings but do not seem to have reached extensive success. Control by vaccination is an alternative that has been longtime in use in some regions and species. A substantial number of vaccination studies where objective information is amenable to meta-analysis treatment have been published in peer reviewed journals or in conference proceedings or other media. The high heterogeneity among reports makes it difficult to accept that the narrow statistical confidence interval obtained in these meta-analyses actually represents the true range of reduction in the whole set of trials. However, the results analyzed here clearly show a general positive effect from vaccination, negative effects only in a few trials, and a positive average balance according to all three variables considered (production, epidemiological or pathogenetic effects). In terms of quantitative reduction, the minimum is an 11% reduction in MAP isolation, which could be considered the worst case average, but with a common outcome at over 50% which is highly profitable from a production point of view. This strategy thus has high chances of have a effect on the overall environmental contamination with MAP, which would mean a significant advance in the fight against paratuberculosis, both in the animal and in the (potential) human public health fields.

## Abbreviations

CC: Clinical Cases; CWC: Cell Wall Competent; CWD: Cell Wall Deficient; E: Experimental infection; F: Field trial; FC: Fecal culture; GL: Gross lesions; HL: Histological lesions; IBD: Inflammatory Bowel Disease; IF: Comparison of initial versus final prevalence; MAP: Mycobacterium avium subsp. paratuberculosis; MC: Comparison to matched controls; NCV: Non-commercial vaccine; NVH: Comparison to non-vaccinating herds; PCR: Polymerase Chain Reaction; PPDbov: Purified Protein Derivative from Mycobacterium bovis; PPDav: Purified Protein Derivative from Mycobacterium avium subsp. avium; P/O/P: Paraffin, Olive Oil, Pumice Stone Powder; Rec: Recombinant; TC: Tissue culture; TM: Total mortality, PCR: Polymerase Chain Reaction, MAP: Mycobacterium avium subsp. Paratuberculosis; IBD: Inflammatory Bowel Disease.

## Competing interests

Felix Bastida works for Vacunek, a small animal health biotechnology company. He is currently working on the development of a new paratuberculosis vaccine for cattle in collaboration with NEIKER and CZ Veterinaria, the producer of Gudair^®^, a commercial paratuberculosis vaccine for use in sheep and goats.

Ramon A. Juste works for a Regional Government funded Agricultural Research Institute that receives funding for research projects from local, regional, national and European Governments, as well as, from companies such as CZ Veterinaria and Vacunek.

## Authors' contributions

RAJ conceived of the study and performed the statistical analysis. Both authors (FB and RAJ) participated in the design of the study, acquisition of data and helped to draft the manuscript. Both read and approved the final manuscript.

## References

[B1] Chiodini RJ Van Kruiningen HJ Merkal RS Ruminant paratuberculosis (Johne's disease): the current status and future prospects Cornell Vet 1984 74 218 262 6375961

[B2] Chiodini RJ Van Kruiningen HJ Eastern white-tailed deer as a reservoir of ruminant paratuberculosis J Am Vet Med Assoc 1983 182 168 169 6826436

[B3] Manning EJ Mycobacterium avium subspecies paratuberculosis: a review of current knowledge J Zoo Wildl Med 2001 32 293 304 12785677 10.1638/1042-7260(2001)032[0293:MASPAR]2.0.CO;2

[B4] Pickup RW Rhodes G Bull TJ Arnott S Sidi-Boumedine K Hurley M Hermon-Taylor J Mycobacterium avium subsp. paratuberculosis in lake catchments, in river water abstracted for domestic use, and in effluent from domestic sewage treatment works: diverse opportunities for environmental cycling and human exposure Appl Environ Microbiol 2006 72 4067 4077 10.1128/AEM.02490-05 16751517 PMC1489623

[B5] Stop the cull. Animal vaccines prevent disease but founder because of political motivations Nat Biotechnol 2007 25 1329 18066007 10.1038/nbt1207-1329

[B6] Torgerson P Torgerson D Benefits of stemming bovine TB need to be demonstrated Nature 2009 457 657 19194427 10.1038/457657d

[B7] Rossiter CA Burhans WS Farm-specific approach to paratuberculosis (Johne's disease) control Vet Clin North Am Food Anim Pract 1996 12 383 415 8828112 10.1016/s0749-0720(15)30413-8

[B8] Goodger WJ Collins MT Nordlund KV Eisele C Pelletier J Thomas CB Sockett DC Epidemiologic study of on-farm management practices associated with prevalence of Mycobacterium paratuberculosis infections in dairy cattle J Am Vet Med Assoc 1996 208 1877 1881 8675478

[B9] Whittington RJ Marshall DJ Nicholls PJ Marsh IB Reddacliff LA Survival and dormancy of Mycobacterium avium subsp. paratuberculosis in the environment Appl Environ Microbiol 2004 70 2989 3004 10.1128/AEM.70.5.2989-3004.2004 15128561 PMC404446

[B10] Whittington RJ Marsh IB Reddacliff LA Survival of Mycobacterium avium subsp. paratuberculosis in dam water and sediment Appl Environ Microbiol 2005 71 5304 5308 10.1128/AEM.71.9.5304-5308.2005 16151118 PMC1214599

[B11] Muskens J Elbers AR van Weering HJ Noordhuizen JP Herd management practices associated with paratuberculosis seroprevalence in Dutch dairy herds J Vet Med B Infect Dis Vet Public Health 2003 50 372 377 10.1046/j.1439-0450.2003.00697.x 14633206

[B12] Kudahl AB Sorensen JT Nielsen SS Ostergaard S Simulated economic effects of improving the sensitivity of a diagnostic test in paratuberculosis control Prev Vet Med 2007 78 118 129 10.1016/j.prevetmed.2006.10.004 17101188

[B13] Zimmer K Drager KG Klawonn W Hess RG Contribution to the diagnosis of Johne's disease in cattle. Comparative studies on the validity of Ziehl-Neelsen staining, faecal culture and a commercially available DNA-Probe test in detecting Mycobacterium paratuberculosis in faeces from cattle Zentralbl Veterinarmed B 1999 46 137 140 10216457 10.1111/j.0931-1793.1999.00214.x

[B14] Whitlock RH Wells SJ Sweeney RW Van Tiem J ELISA and fecal culture for paratuberculosis (Johne's disease): sensitivity and specificity of each method Vet Microbiol 2000 77 387 398 10.1016/S0378-1135(00)00324-2 11118724

[B15] Aly S Anderson R Gardner I Whitlock R Fyock T Adaska J Comparison of methods for detection of MAP super-shedder cows in a large dairy herd Johnes Disease Integrated Program (JDIP) Annual Meeting; Michigan 2008

[B16] Collins MT Gardner IA Garry FB Roussel AJ Wells SJ Consensus recommendations on diagnostic testing for the detection of paratuberculosis in cattle in the United States J Am Vet Med Assoc 2006 229 1912 1919 10.2460/javma.229.12.1912 17173528

[B17] Alinovi CA Ward MP Lin TL Wu CC Sample handling substantially affects Johne's ELISA Prev Vet Med 2009 90 278 283 10.1016/j.prevetmed.2009.04.004 19477542

[B18] Garrido JM Aduriz G Geijo MV Sevilla I Juste RA Juste RA Comparison of different indirect ELISA methods on reference cattle Proceedings of 7th International Colloquium on Paratuberculosis; June 11-14, 2002; Bilbao, Spain 2002 International Association for Paratuberculosis 52 53

[B19] Dieguez FJ Gonzalez AM Menendez S Vilar MJ Sanjuan ML Yus E Arnaiz I Evaluation of four commercial serum ELISAs for detection of Mycobacterium avium subsp. paratuberculosis infection in dairy cows Vet J 2009 180 231 235 10.1016/j.tvjl.2007.11.004 18314355

[B20] Alinovi CA Ward MP Lin TL Moore GE Wu CC Real-time PCR, compared to liquid and solid culture media and ELISA, for the detection of Mycobacterium avium ssp. paratuberculosis Vet Microbiol 2009 136 177 179 10.1016/j.vetmic.2008.10.012 19091493

[B21] Wells SJ Collins MT Faaberg KS Wees C Tavornpanich S Petrini KR Collins JE Cernicchiaro N Whitlock RH Evaluation of a rapid fecal PCR test for detection of Mycobacterium avium subsp. paratuberculosis in dairy cattle Clin Vaccine Immunol 2006 13 1125 1130 10.1128/CVI.00236-06 16928884 PMC1595318

[B22] Bannantine JP Baechler E Zhang Q Li L Kapur V Genome scale comparison of Mycobacterium avium subsp. paratuberculosis with Mycobacterium avium subsp. avium reveals potential diagnostic sequences J Clin Microbiol 2002 40 1303 1310 10.1128/JCM.40.4.1303-1310.2002 11923349 PMC140397

[B23] Poupart P Coene M Van Heuverswyn H Cocito C Preparation of a specific RNA probe for detection of Mycobacterium paratuberculosis and diagnosis of Johne's disease J Clin Microbiol 1993 31 1601 1605 8315002 10.1128/jcm.31.6.1601-1605.1993 PMC265585

[B24] Irenge LM Walravens K Govaerts M Godfroid J Rosseels V Huygen K Gala JL Development and validation of a triplex real-time PCR for rapid detection and specific identification of M. avium sub sp. paratuberculosis in faecal samples Vet Microbiol 2009 136 166 172 10.1016/j.vetmic.2008.09.087 19095382

[B25] Lu Z Mitchell RM Smith RL Van Kessel JS Chapagain PP Schukken YH Grohn YT The importance of culling in Johne's disease control J Theor Biol 2008 254 135 146 10.1016/j.jtbi.2008.05.008 18573505

[B26] Saxegaard F Fodstad FH Control of paratuberculosis (Johne's disease) in goats by vaccination Vet Rec 1985 116 439 441 10.1136/vr.116.16.439 4002570

[B27] Fridriksdottir V Gunnarsson E Sigurdarson S Gudmundsdottir KB Paratuberculosis in Iceland: epidemiology and control measures, past and present Vet Microbiol 2000 77 263 267 10.1016/S0378-1135(00)00311-4 11118711

[B28] Barry C Corbett D Bakker D Andersen P McNair J Strain S The Effect of Mycobacterium avium Complex Infections on Routine Mycobacterium bovis Diagnostic Tests Vet Med Int 2011 145092 10.4061/2011/145092 PMC3134953 21772961

[B29] Eppleston J Windsor PA Lesions attributed to vaccination of sheep with Gudair for the control of ovine paratuberculosis: post farm economic impacts at slaughter Aust Vet J 2007 85 129 133 10.1111/j.0005-0423.2007.00135.x 17397381

[B30] Chiodini R Age resistance and the reticular groove reflex: link or coincidence? The Paratuberculosis Newsletter 1993 5 29 30

[B31] Perez Perez V Estudio de la paratuberculosis en la especie ovina 1993 University of Zaragoza, Spain

[B32] Chavez Gris G Estudio comparativo de las lesiones y de la respuesta inmunologica observada en corderos infectados experimentalmente con *Mycobacterium paratuberculosis *y de *Mycobacterium avium *sp. *silvaticum* 1993 University of Zaragoza, Spain

[B33] Stabel JR Palmer MV Harris B Plattner B Hostetter J Robbe-Austerman S Pathogenesis of Mycobacterium avium subsp. paratuberculosis in neonatal calves after oral or intraperitoneal experimental infection Vet Microbiol 2009 136 306 313 10.1016/j.vetmic.2008.11.025 19135813

[B34] Reddacliff L Eppleston J Windsor P Whittington R Jones S Efficacy of a killed vaccine for the control of paratuberculosis in Australian sheep flocks Vet Microbiol 2006 115 77 90 10.1016/j.vetmic.2005.12.021 16459030

[B35] Benedictus A Mitchell RM Linde-Widmann M Sweeney R Fyock T Schukken YH Whitlock RH Transmission parameters of Mycobacterium avium subspecies paratuberculosis infections in a dairy herd going through a control program Prev Vet Med 2008 83 215 227 10.1016/j.prevetmed.2007.07.008 17868937

[B36] Perez V Tellechea J Corpa JM Gutierrez M Garcia Marin JF Relation between pathologic findings and cellular immune responses in sheep with naturally acquired paratuberculosis Am J Vet Res 1999 60 123 127 9918160

[B37] van Schaik G Kalis CH Benedictus G Dijkhuizen AA Huirne RB Cost-benefit analysis of vaccination against paratuberculosis in dairy cattle Vet Rec 1996 139 624 627 9123788

[B38] Corpa JM Perez V Sanchez MA Marin JF Control of paratuberculosis (Johne's disease) in goats by vaccination of adult animals Vet Rec 2000 146 195 196 10.1136/vr.146.7.195 10718594

[B39] Koets A Hoek A Langelaar M Overdijk M Santema W Franken P Eden W Rutten V Mycobacterial 70 kD heat-shock protein is an effective subunit vaccine against bovine paratuberculosis Vaccine 2006 24 2550 2559 10.1016/j.vaccine.2005.12.019 16417949

[B40] Kathaperumal K Kumanan V McDonough S Chen LH Park SU Moreira MA Akey B Huntley J Chang CF Chang YF Evaluation of immune responses and protective efficacy in a goat model following immunization with a coctail of recombinant antigens and a polyprotein of Mycobacterium avium subsp. paratuberculosis Vaccine 2009 27 123 135 10.1016/j.vaccine.2008.10.019 18955101

[B41] Velaz-Faircloth M Cobb AJ Horstman AL Henry SC Frothingham R Protection against Mycobacterium avium by DNA vaccines expressing mycobacterial antigens as fusion proteins with green fluorescent protein Infect Immun 1999 67 4243 4250 10417198 10.1128/iai.67.8.4243-4250.1999 PMC96731

[B42] Sechi LA Mara L Cappai P Frothingam R Ortu S Leoni A Ahmed N Zanetti S Immunization with DNA vaccines encoding different mycobacterial antigens elicits a Th1 type immune response in lambs and protects against Mycobacterium avium subspecies paratuberculosis infection Vaccine 2006 24 229 235 10.1016/j.vaccine.2005.08.086 16183174

[B43] Kadam M Shardul S Bhagath JL Tiwari V Prasad N Goswami PP Coexpression of 16.8 kDa antigen of Mycobacterium avium paratuberculosis and murine gamma interferon in a bicistronic vector and studies on its potential as DNA vaccine Vet Res Commun 2009 33 597 610 10.1007/s11259-009-9207-6 19199070

[B44] Roupie V Leroy B Rosseels V Piersoel V Noel-Georis I Romano M Govaerts M Letesson JJ Wattiez R Huygen K Immunogenicity and protective efficacy of DNA vaccines encoding MAP0586c and MAP4308c of Mycobacterium avium subsp. paratuberculosis secretome Vaccine 2008 26 4783 4794 10.1016/j.vaccine.2008.07.009 18652866

[B45] Park SU Kathaperumal K McDonough S Akey B Huntley J Bannantine JP Chang YF Immunization with a DNA vaccine cocktail induces a Th1 response and protects mice against Mycobacterium avium subsp. paratuberculosis challenge Vaccine 2008 26 4329 4337 10.1016/j.vaccine.2008.06.016 18582521

[B46] Bull TJ Gilbert SC Sridhar S Linedale R Dierkes N Sidi-Boumedine K Hermon-Taylor J A novel multi-antigen virally vectored vaccine against Mycobacterium avium subspecies paratuberculosis PLoS ONE 2007 2 e1229 10.1371/journal.pone.0001229 18043737 PMC2082073

[B47] Huntley JF Stabel JR Paustian ML Reinhardt TA Bannantine JP Expression library immunization confers protection against Mycobacterium avium subsp. paratuberculosis infection Infect Immun 2005 73 6877 6884 10.1128/IAI.73.10.6877-6884.2005 16177367 PMC1230947

[B48] Singh SV Singh PK Singh AV Sohal JS Gupta VK Vihan VS Comparative efficacy of an indigenous 'inactivated vaccine' using highly pathogenic field strain of Mycobacterium avium subspecies paratuberculosis 'Bison type' with a commercial vaccine for the control of Capri-paratuberculosis in India Vaccine 2007 25 7102 7110 10.1016/j.vaccine.2007.07.054 17804124

[B49] Juste RA Alonso-Hearn M Molina E Geijo M Vazquez P Sevilla IA Garrido JM Significant reduction in bacterial shedding and improvement in milk production in dairy farms after the use of a new inactivated paratuberculosis vaccine in a field trial BMC Res Notes 2009 2 233 10.1186/1756-0500-2-233 19930604 PMC2788577

[B50] Windsor P Research into vaccination against ovine Johne's disease in Australia Small Ruminant Research 2006 62 139 142 10.1016/j.smallrumres.2005.07.044

[B51] Emery DL Whittington RJ An evaluation of mycophage therapy, chemotherapy and vaccination for control of Mycobacterium avium subsp. paratuberculosis infection Vet Microbiol 2004 104 143 155 10.1016/j.vetmic.2004.08.014 15564023

[B52] Rosseels V Huygen K Vaccination against paratuberculosis Expert Rev Vaccines 2008 7 817 832 10.1586/14760584.7.6.817 18665779

[B53] Vallee H R P Etudes sur l'enterite paratuberculeuse des bovides (note preliminaire) Rev Gen Med Vet 1926 35 1 9

[B54] Sigurdsson B A killed vaccine against paratuberculosis (Johne's disease) in sheep Am J Vet Res 1960 21 54 67 14446609

[B55] Domenech JM Massons I Bioestadística Métodos Estadísticos Para Investigadores Temas Fundamentales de Psicología 1982 4 Barcelona

[B56] Chiodini RJ Abolish Mycobacterium paratuberculosis strain 18 J Clin Microbiol 1993 31 1956 1958 8123098 10.1128/jcm.31.7.1956-1958.1993 PMC265672

[B57] Begg DJ Griffin JF Vaccination of sheep against M. paratuberculosis: immune parameters and protective efficacy Vaccine 2005 23 4999 5008 10.1016/j.vaccine.2005.05.031 15992970

[B58] Uzonna JE Chilton P Whitlock RH Habecker PL Scott P Sweeney RW Efficacy of commercial and field-strain Mycobacterium paratuberculosis vaccinations with recombinant IL-12 in a bovine experimental infection model Vaccine 2003 21 3101 3109 10.1016/S0264-410X(03)00261-5 12804836

[B59] Benedictus G Dinkla ETB Wentink GH Thorel MF, Merkal RS Preliminary results of vaccination against paratuberculosis in adult dairy cattle Proceedings International Colloquium on Paratuberculosis, II; Laboratoire Central de Recherches Veterinaires, Maisons-Alfort, France 1988 International Association for Paratuberculosis 136 140

[B60] Juste RA Casal J An economic and epidemiologic simulation of different control strategies for ovine paratuberculosis Prev Vet Med 1993 15 101 115 10.1016/0167-5877(93)90106-4

[B61] Argenté G Thorel MF, Merkal RS Utilisation de la culture fecale dans un plan de prevention de la paratuberculose dans 500 tropeaux; Justifications techniques et economiques Proceedings of the International Colloquium on Paratuberculosis, II; Laboratoire Central de Recherches Veterinaires. Maisons-Alfort. France 1988 International Association for Paratuberculosis 30 35

[B62] Vordermeier M Gordon SV Hewinson RG Mycobacterium bovis antigens for the differential diagnosis of vaccinated and infected cattle Vet Microbiol 2011 151 8 13 10.1016/j.vetmic.2011.02.020 21411245

[B63] Possible links between Crohn's disease and Paratuberculosis Book Possible links between Crohn's disease and Paratuberculosis 2000 SANCO/B3/R16/2000. EUROPEAN COMMISSION DIRECTORATE-GENERAL HEALTH & CONSUMER PROTECTION Directorate B - Scientific Health Opinions Unit B3 - Management of scientific committees II

[B64] Juste RA Geijo MV Sevilla I Aduriz G Garrido JM Juste RA Control of paratuberculosis by vaccination Proceedings of the 7th International Colloquium on Paratuberculosis; Bilbao, Spain 2002 International Association for Paratuberculosis 331

[B65] Hagan WA Vaccination against Johne's disease Cornell Vet 1935 25 344 353

[B66] Doyle TM I.A. SAWaG Johne's disease Infectious Diseases of Animals 1959 1 London: Butterworth's Scientific Publications 319 345

[B67] Doyle TM Vaccination against Johne's disease Vet Rec 1964 76 73 76

[B68] Stuart P Vaccination against Johne's Disease in Cattle Exposed to Experimental Infection Br Vet J 1965 121 289 318 14344190 10.1016/s0007-1935(17)41102-x

[B69] Wilesmith JW Johne's disease: a retrospective study of vaccinated herds in Great Britain Br Vet J 1982 138 321 331 7116121 10.1016/s0007-1935(17)31037-0

[B70] Hurley S Ewing E Merkal RS Results of a field evaluation of a whole cell bacterin Proceedings of the International Colloquium on Paratuberculosis, I; NADC, USDA, Ames, IA, USA 1983 International Association for Paratuberculosis 244 24811329228

[B71] Kalis CHJ Benedictus G van Weering HJ Flamand F Haagsma J Chiodini RJ, Kreeger JM Experiences with the use of an experimental vaccine in the control of paratuberculosis in The Netherlands Proceedings of the 3rd International Colloquium on Paratuberculosis; Providence, RI, USA 1992 International Association for Paratuberculosis 484 494

[B72] Wentink GH Bongers JH Zeeuwen AA Jaartsveld FH Incidence of paratuberculosis after vaccination against M. paratuberculosis in two infected dairy herds Zentralbl Veterinarmed B 1994 41 517 522 7701865 10.1111/j.1439-0450.1994.tb00258.x

[B73] Xenos G Yiannati A Dimarelli Z Mtliangas P Koutsoukou E PO Evaluation of a live paratuberculosis vaccine in sheep and goats CEC Workshop Commission of the Economic Communities; Crete, Greece 1988

[B74] Aduriz JJ Epidemiología, diagnóstico y control de la paratuberculosis ovina en la Comunidad Autónoma del País Vasco 1993 University of Zaragoza, Spain

[B75] Aduriz JJ Juste RA Sáez de Ocáriz C Chiodini RJ, Collins MT, Bassey EOE An epidemiologic study of sheep paratuberculosis in the Basque Country of Spain: serology and productive data Proceedings of the 4th International Colloquium on Paratuberculosis; Rehoboth, MA, USA 1995 International Association for Paratuberculosis 19 26

[B76] Cranwell MP Control of Johne's disease in a flock of sheep by vaccination Vet Rec 1993 133 219 220 10.1136/vr.133.9.219 8236728

[B77] Pérez V García Marín JF Bru R Moreno B JJ B Resultados obtenidos en la vacunación de ovinos adultos frente a paratuberculosis Med Vet 1995 12 196 201

[B78] Gwozdz JM Thompson KG Manktelow BW Murray A West DM Vaccination against paratuberculosis of lambs already infected experimentally with Mycobacterium avium subspecies paratuberculosis Aust Vet J 2000 78 560 566 10.1111/j.1751-0813.2000.tb11902.x 10979513

[B79] Windsor PA Eppleston J Sergeant E Monitoring the efficacy of Gudair™ OJD vaccine in Australia Proc Aust Sheep Vet Soc 2003 114 122

[B80] Eppleston J Reddacliff L Windsor P Whittington R Jonbes S Field studies on vaccination for the control of OJD in Australia - and overview Proc Aust Sheep Vet Soc 2004 56 59

[B81] Griffin JF Hughes AD Liggett S Farquhar PA Mackintosh CG Bakker D Efficacy of novel lipid-formulated whole bacterial cell vaccines against Mycobacterium avium subsp. paratuberculosis in sheep Vaccine 2009 27 911 918 10.1016/j.vaccine.2008.11.053 19059295

[B82] Hore DE McQueen DS McKinna DA Infection of dairy cattle with Mycobacterium johnei in a partially vaccinated herd Aust Vet J 1971 47 421 423 10.1111/j.1751-0813.1971.tb02169.x 5166580

[B83] Larsen AB Merkal RS Moon HW Evaluation of a paratuberculosis vaccine given to calves before infection Am J Vet Res 1974 35 367 369 4819719

[B84] Jorgensen JB Merkal RS The effect of vaccination on the excretion of Mycobacterium paratuberculosis Proceedings of the International Colloquium on Paratuberculosis, I; NADC, USDA, Ames IA, USA 1983 International Association for Paratuberculosis 249 25411329228

[B85] Argenté G Chiodini RJ, Kreegel JM Efficiency of vaccination and other control measures estimated by fecal culturing in a regional program Proceedings of the 3rd International Colloquium on Paratuberculosis; Orlando, Florida, USA 1992 International Association for Paratuberculosis 495 503

[B86] Kormendy B The effect of vaccination on the prevalence of paratuberculosis in large dairy herds Vet Microbiol 1994 41 117 125 10.1016/0378-1135(94)90141-4 7801515

[B87] Mohr P Johne's can be managed Johne's can be managed 2000 16 1722040359

[B88] Kalis CH Hesselink JW Barkema HW Collins MT Use of long-term vaccination with a killed vaccine to prevent fecal shedding of Mycobacterium avium subsp paratuberculosis in dairy herds Am J Vet Res 2001 62 270 274 10.2460/ajvr.2001.62.270 11212038

[B89] Klawonn W Cussler K Drager KG Gyra H Kohler H Zimmer K Hess RG [The importance of allergic skin test with Johnin, antibody ELISA, cultural fecal test as well as vaccination for the sanitation of three chronically paratuberculosis-infected dairy herds in Rhineland-Palatinate] Dtsch Tierarztl Wochenschr 2002 109 510 516 12596564

[B90] Muñoz M García Marín JF García-Pariente C Reyes LE Verna A Moreno O Fuertes M Doce J Puentes E Garrido J Pérez V Manning EJB, Nielsen SS Efficacy of a killed vaccine (SILIRUM) in calves challenged with MAP Proceedings of 8th International Colloquium on Paratuberculosis; Copenhagen, Denmark 2005 International Association for Paratuberculosis 208 217

[B91] Patton E Konkle D Fish R Engrav J Bohn J Role of vaccination in the control of Johne's disease in 3 Wisconsin dairy herds Book Role of vaccination in the control of Johne's disease in 3 Wisconsin dairy herds 2006 City: Wisconsin Department of Agriculture, Trade & Consumer Protection, Division of Animal Health

[B92] Kathaperumal K Park SU McDonough S Stehman S Akey B Huntley J Wong S Chang CF Chang YF Vaccination with recombinant Mycobacterium avium subsp. paratuberculosis proteins induces differential immune responses and protects calves against infection by oral challenge Vaccine 2008 26 1652 1663 10.1016/j.vaccine.2008.01.015 18304707

[B93] Sweeney RW Whitlock RH Bowersock TL Cleary DL Meinert TR Habecker PL Pruitt GW Effect of subcutaneous administration of a killed Mycobacterium avium subsp paratuberculosis vaccine on colonization of tissues following oral exposure to the organism in calves Am J Vet Res 2009 70 493 497 10.2460/ajvr.70.4.493 19335105

[B94] Brotherston JG Gilmour NJL Quantitative studies of *Mycobacterium johnei *in the tissues of sheep. I Routes of infection and assay of viable *M. johnei* J Comp Pathol 1961 71 286 299

[B95] Nisbet DI Gilmour NJ Brotherston JG Quantitative studies of Mycobacterium johnei in tissues of sheep. III. Intestinal histopathology J Comp Pathol 1962 72 80 91 14479935 10.1016/s0368-1742(62)80009-5

[B96] Juste RA Garcia Marin JF Peris B Saez de Ocariz CS Badiola JJ Experimental infection of vaccinated and non-vaccinated lambs with Mycobacterium paratuberculosis J Comp Pathol 1994 110 185 194 10.1016/S0021-9975(08)80189-2 8040384

[B97] Dimareli-Malli Z Sarris K Papadopoulos O N I Xenos G A M Papadopoulos G Evaluation of an inactivated whole cell experimental vaccine against paratuberculosis in sheep and goats PTBC Newsletter 1997 9 10 17

[B98] Eppleston J Reddacliff L Windsor P Links I Whittington R Preliminary observations on the prevalence of sheep shedding Mycobacterium avium subsp paratuberculosis after 3 years of a vaccination program for ovine Johne's disease Aust Vet J 2005 83 637 638 10.1111/j.1751-0813.2005.tb13279.x 16255289

[B99] Toribio JA Sergeant ES A comparison of methods to estimate the prevalence of ovine Johne's infection from pooled faecal samples Aust Vet J 2007 85 317 324 10.1111/j.1751-0813.2007.00188.x 17685977

[B100] Marly J Thorel MF Perrin GG Pardon P Guerrault PJ Thorel MF, Merkal RS Suivi de vaccination de chevrettes contre la paratuberculose: Consequences cliniques, serologiques et allergiques et epreuve virulente Proceedings of the International Colloquium on Paratuberculosis, II; Laboratoire Central de Recherches Veterinaires. Maisons-Alfort. France 1988 International Association for Paratuberculosis 99 109

[B101] Leslie P Riemann HP West G Moe A Thorel MF, Merkal RS Vaccination field trial for Mycobacterium paratuberculosis (Johne's disease) in the caprine species Proceedings of the International Colloquium on Paratuberculosis, II; Laboratoire Central de Recherches Veterinaires. Maisons-Alfort, France 1988 International Association for Paratuberculosis 110 129

[B102] Hines ME Stiver S Giri D Whittington L Watson C Johnson J Musgrove J Pence M Hurley D Baldwin C Efficacy of spheroplastic and cell-wall competent vaccines for Mycobacterium avium subsp. paratuberculosis in experimentally-challenged baby goats Vet Microbiol 2007 120 261 283 10.1016/j.vetmic.2006.10.030 17123751

[B103] García-Pariente C Pérez V Geijo M Moreno O Muñoz M Fuertes M Puentes E Doce J Ferreras MC Garcia Marin JF Manning EJB, Nielsen SS The efficacy of a killed vaccine against paratuberculosis (SILIRUM^®^) in cattle. A field study Proceedings of the 8th International Colloquium on Paratuberculosis; Copenhagen, Denmark 2005 International Association for Paratuberculosis 52

[B104] Sommerville EM Wakelin RL Hutton JB Vaccination of lambs at 3 to 4 months of age protects against Johne's disease PTBC Newsletter 1995 7 25 29

[B105] Reyes LE González J Benavides J Peris Palau B, P. MP, LA M, GM A Nuevos adyuvantes en la vacunación frente a la paratuberculosis ovina XXVII Jornadas Científicas y VI Jornadas Internacionales de la Sociedad Española de Ovinotecnia y Caprinotecnia, SEOC; Spain 2002 SEOC 758 761

[B106] Thonney ML SS Smith MC Control of Johne's disease in sheep by vaccination Preliminary Report Control of Johne's disease in sheep by vaccination Preliminary Report 2005 Cornell University22004739

